# Cardiovascular and metabolic issues in the treatment of schizophrenia: focus on the management of negative symptoms

**DOI:** 10.1192/j.eurpsy.2024.74

**Published:** 2024-08-27

**Authors:** P. Falkai

**Affiliations:** Psychiatry, University of Munich, Munich, Germany

## Abstract

**Abstract:**

Mortality from cardiovascular disease is increased in people with mental health disorders in general and schizophrenia in particular. The causes are multifactorial, but it is known that antipsychotic medication can cause cardiac side-effects beyond the traditional coronary risk factors. Schizophrenia itself is a contributor to an increased risk of cardiovascular mortality via cardiac autonomic dysfunction and a higher prevalence of metabolic syndrome, both contributing to a reduced life expectancy.

Overall, management of cardiovascular risk within this population group must be multifaceted and nuanced to allow the most effective treatment of serious mental illness to be conducted within acceptable parameters of cardiovascular risk; some practical measures are presented for the clinical cardiologist.

**Disclosure of Interest:**

None Declared

